# Tuberculosis among health care workers in KwaZulu-Natal, South Africa: a retrospective cohort analysis

**DOI:** 10.1186/1471-2458-14-891

**Published:** 2014-08-30

**Authors:** Carrie Tudor, Martie Van der Walt, Bruce Margot, Susan E Dorman, William K Pan, Gayane Yenokyan, Jason E Farley

**Affiliations:** Fogarty Global Health Post-doctoral Fellow, University of North Carolina, Chapel Hill, 211 W. Sierra Circle, San Marcos, TX 78666 USA; South African Medical Research Council, Pretoria, South Africa; KwaZulu-Natal Department of Health, Pietermaritzburg, KwaZulu-Natal, South Africa; Johns Hopkins University School of Medicine, Baltimore, Maryland USA; Duke University Nicholas School of Environment and Duke Global Health Institute, Durham, NC USA; Johns Hopkins University Bloomberg School of Public Health, Baltimore, Maryland USA; Johns Hopkins University School of Nursing, Baltimore, Maryland USA

**Keywords:** Tuberculosis, Health care worker, HIV, Occupational health, Infection control

## Abstract

**Background:**

Tuberculosis (TB) is an occupational hazard for health care workers (HCWs) who are at greater risk of developing TB than the general population. The objective of this study was to compare the difference in TB incidence among HCWs with versus without a history of working in TB wards, to estimate the incidence of TB among HCWs, and to identify risk factors for TB disease in HCWs.

**Methods:**

A retrospective cohort study (January 2006 to December 2010) was conducted in three district hospitals in KwaZulu-Natal, South Africa. Data were abstracted via chart review from occupational health medical records. Bivariate and multivariate analyses were performed using a Poisson multilevel mixed model.

**Results:**

Of 1,313 (92%) medical charts reviewed with data on location of work documented, 112 (9%) cases of TB were identified. Among HCWs with TB 14 (13%) had multidrug-resistant TB. Thirty-six (32%) were cured, 33 (29%) completed treatment, and 13 (12%) died. An increased incidence of TB was reported for HCWs with a history of working in TB wards (incidence rate ratio [IRR] 2.03, 95% CI 1.11-3.71), pediatric wards (IRR 1.82 95% CI 1.07-3.10), outpatient departments (IRR 2.08 95% CI 1.23-3.52), and stores/workshop (IRR 2.38 95% CI 1.06-5.34) compared with those without such a history. HCWs living with HIV had a greater incidence of TB (IRR 3.2, 95% CI 1.54-6.66) than HIV-negative HCWs. TB incidence among HCWs was approximately two-fold greater than that of the general population over the study period.

**Conclusions:**

HCWs working in a TB ward had an increased incidence of TB. However, a greater incidence of TB was also found in HCWs working in other wards including pediatric wards, outpatient departments and stores. We also identified a greater incidence of TB among HCWs than the general population. These findings further support the need for improved infection control measures not only in TB or drug-resistant TB wards or areas perceived to be at high-risk but also throughout hospitals to protect HCWs. Additionally, it is recommended for occupational health services to routinely screen HCWs for TB and provide HCWs with access to care for TB and HIV.

## Background

South Africa had the third highest incident cases of tuberculosis (TB) globally with an annual incidence of 993/100,000 in 2011 [1], and an incidence as high as 1,142/100,000 in KwaZulu-Natal province [2]. TB has long been considered an occupational hazard for health care workers (HCWs) [3, 4] and it is estimated that the risk of active TB disease is two to three times greater in HCWs than in the general population [5, 6]. More than 50% of HCWs worldwide are estimated to have latent TB infection; however the burden of active TB disease among HCWs is less well documented [5, 7]. The overwhelming majority of cases of TB among HCWs occur in low- and middle-income countries. It is generally believed that hospital-associated transmission of TB from patient to HCW is the most likely mode of transmission due to poor or non-existent infection control measures [5].

The high rates of TB in South Africa are closely linked with the high rates of human immunodeficiency virus (HIV) in the country, which has one of the highest prevalence estimates of HIV globally, at 17.8% [8]. South Africa has one of the highest TB/HIV co-infection rates estimated to be 65% in 2011 [1] and this rate is even greater in KwaZulu-Natal where 80% of TB cases are co-infected with HIV [9]. Results of surveys among HCWs in South Africa have indicated that up to 16% of HCWs in South Africa are living with HIV [10, 11], placing these HCWs at an even greater risk of developing TB in the workplace than HCWs without HIV.

Cases of drug-resistant TB have increased dramatically in recent years particularly in South Africa, which has one of the highest burdens of drug-resistant TB worldwide [12]. Patients with multidrug-resistant TB (MDR-TB) and extensively drug-resistant (XDR- TB) have been admitted to hospitals for extended periods of time, placing HCWs and other patients at risk of infection with drug-resistant TB due to prolonged exposure [9]. A recent study in KwaZulu-Natal, South Africa found that among patients admitted to a drug-resistant TB referral hospital, those who self-identified as a HCW had a five-fold greater incidence of drug-resistant TB than patients not identified as HCWs [13].

The main objective of this study was to compare the difference in TB incidence between HCWs with versus without a history of working in a TB ward. We also sought to describe TB disease and treatment outcomes and estimate the incidence of TB among a sample of HCWs in KwaZulu-Natal, and to identify risk factors for TB disease in HCWs.

## Methods

A retrospective cohort study of hospital-based HCWs was conducted to identify cases of TB, hospital wards or departments where HCWs worked, and associated risk factors.

### Study setting and sample

KwaZulu-Natal is a province in eastern South Africa with a population of 10.3 million accounting for 20% of South Africa’s total population [14]. KwaZulu-Natal has the highest incidence of TB in South Africa [15] and the average HIV prevalence among women attending antenatal services across the three districts where the study was conducted was 41% in 2011 [16]. The province is divided into 11 health districts with 42 district hospitals.

In KwaZulu-Natal province, there are three district general hospitals with a specialized MDR-TB ward located on the grounds of the hospital; the study was conducted in these three hospitals. These hospitals were geographically distributed across the province and were located in rural socio-economically disadvantaged areas with high rates of unemployment [14]. The study hospitals ranged in size from 170 to 280 beds and employed between 332 and 700 HCWs. The majority of the inpatient wards in the study hospitals were open multi-bed wards with limited or no space for isolating patients suspected of TB. None of the hospitals used mechanical ventilation or ultraviolet germicidal irradiation (UVGI). Infection control measures varied by hospital and by wards within hospitals. N95 respirators were not consistently available across all wards and areas of the hospitals.

For the purposes of this study, the World Health Organization (WHO) definition of HCW was used which defines a HCW as any person whose main activities are aimed at enhancing health [17]. This includes clinical staff (doctors, nurses, etc.), paramedical staff (radiographers, laboratory personnel, pharmacists, etc.), support staff (cleaners, orderlies, porters, etc.) and administrative staff.

A TB ward was defined as a ward where patients with drug-susceptible or presumed drug-susceptible TB are admitted and a MDR-TB ward was defined as a separate specialized ward where patients with confirmed MDR-TB are admitted.

Each of the three study hospitals had an occupational health clinic staffed by a nurse. New employees undergo a pre-placement health assessment prior to beginning work in the facility and an occupational health medical record is created. Occupational health clinics provide preventive and treatment services for occupational illnesses and injuries such as HIV post-exposure prophylaxis for needle stick injuries as well as general wellness services (family planning, immunization, etc.), and HIV counseling and testing.

### Data collection

Data on HCWs diagnosed with TB were collected through chart review of employee occupational health medical records at the three study hospitals between July and September 2011. Cases of TB disease among HCWs were identified based on new documented diagnosis during a five-year period between January 1, 2006 and December 31, 2010. The following data were abstracted using a standardized chart audit form: age (at time of abstraction), sex, race, occupation, years working in facility, wards or departments where a HCW worked, HIV-status (at time of abstraction), previous TB history, type of TB, and comorbidities (e.g. diabetes).

Study data were collected on paper forms and entered into a secure password protected database, using REDCap (Research Electronic Data Capture) an electronic data capture tools hosted at the Johns Hopkins University on a secure server [18].

### Statistical analysis

Univariate analysis was first performed on all variables to screen for coding and entry errors and to check for normality and skewness. Bivariate analysis compared characteristics of individuals with the exposure of a history of working in a TB ward versus those without a history of working in a TB ward. Differences between categorical variables were assessed with χ^2^ or Fisher’s exact tests, as appropriate. Student’s *t*-tests were used to compare means of continuous variables.

Annual incidence rates were calculated as the number of new cases of TB among HCWs working per the total number of HCWs employed during that year and the total time at risk expressed as an annual rate per 100,000 HCW population for each hospital. Incidence rates for each of the three districts and the province as a whole were estimated from published TB case finding and population data for each year [2, 19, 20]. Incidence rates of TB among HCWs of the three hospitals and the general population of each district and the province were compared using incidence rate ratios (IRR) with 95% confidence intervals.

A Poisson model with a random effect for individual was developed and fit using a generalized linear latent and mixed model (GLLAMM) in Stata version 11 [21] to assess associations between independent variables and the incidence of TB. Variables included as fixed effects were: age (centered at the mean), duration of employment (duration of employment in the facility minus the total person-time in years), occupation (categorical variable with administrative staff as the reference group), HIV status (categorical with HIV-negative as reference group; HIV-positive, and HIV status not recorded), history of work in a TB ward, general ward, pediatric ward, outpatient department, primary health care center, stores/workshop, and number of wards HCWs had worked in (1 ward vs. >1 ward). Adaptive Gaussian quadrature with 10 points was used to estimate the parameters of the models. Model selection was performed comparing the Akaike information criterion (AIC) and the model with the lowest AIC was selected. Variables were retained in the final model if the p-value was <0.20 in the unadjusted analysis.

### Ethics

This study was reviewed and approved by the Institutional Review Board of the Johns Hopkins Medical Institutions, the Ethics Committee of the South African Medical Research Council and the Provincial Health Research Committee of the KwaZulu-Natal Province Department of Health. In addition, management at each hospital provided letters of approval. Informed consent was waived to access health care worker’s charts and was approved by the ethics committees.

## Results

A total of 1,427 occupational health employee medical records were reviewed across the three hospitals and the completeness of the records varied across the three hospitals: 85% (474/559), 90% (227/251), and 99% (612/617). The hospital with the highest proportion of complete medical charts had a full-time occupational health nurse with postgraduate occupational health training [22]. One hundred fourteen (8%) records did not contain information on where the HCW worked, and these records were excluded from analyses. Of the 1,313 records included in this analysis 1,003/1,313 (78%) of HCWs were female, with a mean age of 40.1 (standard deviation [SD] 10. 2) ranging from 20 to 70 (Table 1). Occupation was collapsed into four main categories: clinical, support, paramedical, and administrative. The majority of HCWs were clinical staff 717 (55%); among these 14 (1%) were doctors, 237 (18%) professional nurses, 211 (16%) enrolled nurses, and 205 (16%) enrolled nursing assistants. Support staff comprised the next largest group of staff 325 (25%) with general orderlies accounting for 59% (n = 192) of this category. Approximately 15% (n = 201) of HCWs worked in more than one ward or area of the hospital, ranging from one to five wards. Clinical and a large proportion of support staff spend considerable time in wards or areas of the hospital with patients and potentially exposed to TB.

**Table 1:** **Health care worker characteristics in three district hospitals in KwaZulu-Natal, South Africa (January 2006 – December 2010) (n = 1,313)**

Variable	Total	Worked in TB ward	Did not work in TB ward	p-value
	n = 1,313	n = 69	n = 1,244	
	n (%)	n (%)	n (%)	
Mean age in years (SD) (n = 1,281)	40.1 (10.2)	39.7 (8.8)	40.1 (10.3)	0.79^*^
	Range 20-70	Range 25-62	Range 20-70	
Sex (n = 1,285)				
Female	1,003 (78)	57 (84)	946 (78)	0.24^**^
Male	282 (22)	11 (16)	271 (22)	
Race (n = 1,307)				
African / black	1264 (97)	69 (100)	1195 (97)	0.48^***^
Asian	22 (2)	0	22 (2)	
White	18 (1)	0	18 (1)	
Coloured	3 (0)	0	3 (0)	
Occupation^^^ (n = 1,307)				
Clinical staff	717(55)	56 (81)	661 (53)	<0.01^***^
Support staff	325 (25)	6 (9)	319 (26)	
Administrative	142 (11)	4 (6)	138 (11)	
Paramedical	123 (9)	3 (4)	120 (10)	
Mean duration (years) working in hospital (n = 1,305)	7.4 (7.2)	7.2 (7.2)	7.4 (7.2)	0.82^*^
	Range 0-40	Range 1-32	Range 0-40	
Wards where HCW worked^†^				
General ward	261 (20)	17 (25)	244 (20)	0.31^**^
Pediatric ward	103 (8)	7 (10)	96 (8)	0.47^**^
Maternity	100 (8)	3 (4)	97 (8)	0.48^***^
MDR-TB ward	78 (6)	6 (9)	72 (6)	0.32^**^
Outpatient department	101 (8)	5 (7)	96 (8)	0.89^**^
Primary health center	106 (8)	4 (6)	102 (8)	0.65^***^
Throughout hospital	159 (12)	1 (2)	158 (13)	<0.01^***^
Casualty	32 (2)	4 (6)	28 (2)	0.08^***^
Number of wards HCW worked in				
> 1 ward	201 (15)	36 (52)	165 (13)	<0.01^**^
Diabetes	56 (4)	3 (4)	53 (4)	1.00^***^
HIV status				
HIV-negative	260 (20)	10 (15)	250 (20)	0.50^**^
HIV-positive	79 (6)	5 (7)	74 (6)	
HIV status not recorded	974 (74)	54 (78)	920 (74)	
Previously treated for TB^‡^ (n = 1,000)	93 (9)	5 (8)	88 (9)	0.76^**^
TB diagnosed between 1 January 2006 and 31 December 2010	112 (9)	14 (20)	98 (8)	<0.01^**^
Pulmonary TB	72 (64)	10 (71)	62 (63)	0.74^***^
Extrapulmonary TB	18 (16)	1 (7)	17 (17)	0.59^***^
Drug-resistant TB	14 (13)	0 (0.0)	14 (14)	0.31^***^

^*^Student’s *t*-test, SD = standard deviation.

^**^χ^2^.

^***^Fisher’s exact test.

^^^Administrative staff includes finance, human resources, management) data capturers, office clerks and ward clerks; paramedical staff includes lay counselors, radiographers, pharmacists, lab technicians, paramedics, mortuary attendants, dieticians, social workers, physiotherapists, occupational therapists, and speech therapists; support staff includes orderlies, cleaners, groundsman, maintenance, kitchen staff, laundry, and security; and clinical staff includes medical doctors/medical officers, nurses (professional nurse, enrolled nurse, nursing assistant and student nurse) and dentists.

^†^Not mutually exclusive – health care workers worked in more than one ward.

^‡^TB prior to 2006, prior to starting work in one of the study hospitals, or TB after 2010.

HCW characteristics by exposure of history of working in a TB ward are presented in Table 1. There was no difference between the two exposure groups on demographic characteristics except for occupation. Those with a history of working in a TB ward were more likely to work in more than one ward 52% (n = 36) than those without a history of working in a TB ward 13% (n = 165) (p < 0.01).

### TB among health care workers

Among the 1,313 HCWs whose records were included in the analysis, 112 (9%) cases of TB were identified during the five-year period of interest. A greater proportion of HCWs 20% (n = 14) that worked in a TB ward had TB compared with HCWs without a history of working in a TB ward 8% (n = 98) (p < 0.01). HCWs who worked in one ward were less likely to develop TB 8% (n = 83) than those who worked in more than one ward 15% (n = 29) (p < 0.01). Among HCWs diagnosed with TB, the largest category 48% (n = 53) were clinical staff (all nursing staff) followed by support staff 33% (n = 36), and female 70% (n = 78). Of the 112 cases of TB 28 (25%) did not have information on type of TB recorded in their chart. Table 2 describes TB cases among HCWs who had type of TB (drug-susceptible or drug-resistant) documented in their chart (n = 84). Fourteen (13%) HCWs had drug-resistant TB, among which 13 (93%) had MDR-TB and 1 (7%) XDR-TB. None of the 14 drug-resistant TB cases occurred in HCWs with a history of working in a TB or MDR-TB ward. The largest category of cases 44 (52%) were primary cases, including six MDR-TB and one XDR-TB case.

**Table 2:** **Description of drug-susceptible and drug-resistant TB cases among HCWs in KwaZulu-Natal, South Africa (January 2006 - December 2010) (n = 84)**

Variable	Total	HCWs with drug-susceptible TB	HCWs with drug-resistant TB ^*^	p-value
	(n = 84)	n = 70	n = 14	
	n (%)	n (%)	n (%)	
Mean age in years (SD) (n = 75)	40.5 (9.0)	41.0 (9.4)	38.6 (7.1)	0.37
		Range 25-62	Range 26-52	
Sex				0.52
Female	64 (76)	53 (76)	11 (79)	
TB cases by occupation^**^ (n = 83)				
Clinical	43 (52)	34 (49)	9 (64)	0.72
Support staff	24 (29)	21 (30)	3 (21)	
Paramedical	10 (12)	8 (12)	2 (14)	
Administrative	6 (7)	6 (9)	0	
Work exposure/place of employment^***^				
Outpatient department/casualty/PHC	23 (27)	19 (27)	4 (29)	1.00
General ward	24 (29)	21 (30)	3 (21)	0.75
Non-clinical area^†^	13 (15)	12 (17)	1 (7)	0.69
Pediatric ward	16 (19)	14 (20)	2 (14)	1.00
TB ward/MDR-TB ward	12 (14)	12 (17)	0	0.20
Maternity ward	7 (8)	3 (4)	4 (29)	0.01
Throughout the hospital	8 (10)	7 (10)	1 (7)	1.00
Operating theater	5 (6)	4 (6)	1 (7)	1.00
HIV status				
HIV-negative	13 (15)	10 (14)	3 (21)	0.26
HIV-positive	20 (24)	19 (27)	1 (7)	
HIV status not recorded	51 (61)	41 (59)	10 (71)	
Previous history of TB				
No	44 (52)	37 (53)	7 (50)	1.00
Yes	12 (14)	10 (14)	2 (14)	
Not recorded in chart	28 (33)	23 (33)	5 (36)	

^*^13 MDR-TB cases and 1 XDR-TB case.

^**^Administrative staff includes finance, human resources, management, data capturers, office clerks and ward clerks; paramedical staff includes lay counselors, radiographers, pharmacists, lab technicians, paramedics, mortuary attendants, dieticians, social workers, physiotherapists, occupational therapists, and speech therapists; support staff includes orderlies, cleaners, groundsman, maintenance, kitchen staff, laundry, and security; and clinical staff includes medical doctors/medical officers, nursing staff, and dentists.

^***^Not mutually exclusive – health care workers worked in more than one ward.

^†^Non-clinical area includes laboratory, kitchen, laundry, administrative offices, stores/workshop, mortuary, outside, pharmacy, driving, and therapy.

Of the 112 HCWs with TB, 38 (34%) had their HIV-status documented in their health record. Twenty-four (21%) were HIV-positive, 14 (13%) were HIV-negative, and for 74 (66%) HIV status was not recorded.

### Treatment outcomes

Thirty-two percent (n = 36) of HCWs with TB were cured, 29% (n = 33) completed treatment, and two HCWs (2%) defaulted treatment. Twelve percent (n = 13) of HCWs with TB died: among them four (31%) died with MDR-TB, five (38%) with drug-susceptible TB, and four (31%) did not have TB drug susceptibility recorded in the chart. Twenty-five (22%) HCWs did not have a final treatment outcome recorded in their occupational health medical record; 11 (16%) with drug-susceptible TB, 3 (21%) with MDR-TB and 11 (39) without drug-susceptibility recorded in the chart.

### Incidence rates of TB among HCWs

**Figure 1:**
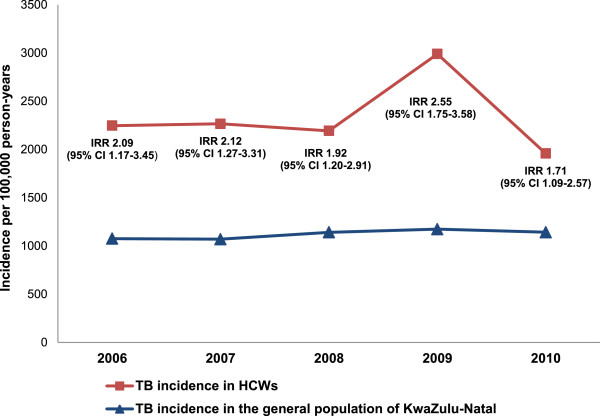
**TB incidence rates among HCWs per 100,000 person-years in KwaZulu-Natal, South Africa (2006-2010).**

The estimated TB incidence rate in HCWs in this sample in 2010 was 1,958/100,000 person-years, which was nearly double the incidence of TB in South Africa (981/100,000) for the same year [12]. As presented in Figure 1 the incidence of TB among HCWs was consistently higher than the incidence of the general population in KwaZulu-Natal from 2006 – 2010.

### Multivariate analysis

HCWs with a history of working in a TB ward, the main exposure of interest, had a nearly three-fold increased incidence of TB (IRR 2.87, 95% CI 1.67–4.93) in the unadjusted analysis, and this association remained significant in the adjusted analysis controlling for other factors (IRR 2.03, 95% CI 1.11-3.71) (Table 3). HCWs with a history of working in other areas such as outpatient departments (IRR 2.08, 95% CI 1.23-3.52), pediatric wards (IRR 1.82, 95% CI 1.07-3.10), and in non-clinical areas, such as the stores department (IRR 2.38, 95% CI 1.06-5.34) also had an increased incidence of TB. Interestingly, HCWs with a history of work in an MDR-TB ward had a lower incidence of TB than individuals without a history of work in an MDR-TB ward, although the difference did not reach statistical significance (IRR 0.34, 95% 0.08 – 1.51). HCWs living with HIV had a three-fold greater incidence of TB compared with HIV-negative HCWs (IRR 3.20, 95% CI 1.54-6.66). We found no significant difference in the incidence of TB between different occupations among this sample.

**Table 3:** **Multivariate multilevel mixed model for the incidence of TB among HCWs working in three public hospitals in KwaZulu-Natal, South Africa (January 2006 – December 2010) (n = 1,313)**

Variable*	Unadjusted IRR (95% CI)	Adjusted IRR (95% CI)
Ward where HCW worked^**^		
TB ward	**2.87 (1.67 – 4.93)**	**2.03 (1.11 – 3.71)**
General ward	**1.73 (1.15 – 2.60)**	1.50 (0.93 – 2.44)
Pediatric ward	**2.70 (1.68 – 4.34)**	**1.82 (1.07 – 3.10)**
Outpatient department	**1.99 (1.17– 3.40)**	**2.08 (1.23 – 3.52)**
Primary health care center	**2.15 (1.18 – 3.89)**	1.49 (0.76 – 2.91)
Stores/workshop	1.95 (0.92 – 4.13)	**2.38 (1.06 – 5.34)**
MDR-TB ward	0.25 (0.06 – 1.08)	0.34 (0.08 – 1.51)
Wards worked in		
1 ward	Ref	Ref
> 1 ward	**2.03 (1.37 – 3.02)**	1.40 (0.84 – 2.34)
Age in years	0.99 (0.97 – 1.01)	0.97 (0.94 – 1.00)
Years worked in hospital	1.01 (0.98 – 1.03)	1.02 (0.99 – 1.06)
HIV status		
HIV-negative	Ref	Ref
HIV-positive	**5.35 (2.58 – 11.10)**	**3.20 (1.54 – 6.66)**
HIV status not recorded	1.16 (0.67 – 2.01)	0.89 (0.52 – 1.55)
Occupation		
Administrative	Ref	Ref
Paramedical staff	2.39 (0.93 – 6.14)	2.25 (0.80 – 6.29)
Support staff	1.81 (0.81 – 4.02)	2.24 (0.95– 5.29)
Clinical staff	1.64 (0.76 – 3.51)	1.43 (0.61 – 3.32)

^*^Variables included as fixed effects were: history of work in a TB ward, general ward, pediatric ward, outpatient department, primary health care center, stores/workshop, number of wards HCWs had worked in (1 ward vs. >1 ward), age (centered at the mean age of 40 years), duration of employment (continuous variable calculated as the duration of employment in the facility minus the total person-time in years, HIV status, and occupation.

^**^Referent group for each ward is no history of working in that ward.

Bold indicates significant findings at p-value <0.05.

## Discussion

Health care workers with an exposure of working in a TB ward had a two-fold greater incidence of TB compared with those without the exposure of working in a TB ward in this study. However, findings from this study also indicated that HCWs who worked in other areas of the hospital including non-clinical areas, such as the stores department, also had a greater incidence of TB. This suggests that TB transmission may be occurring throughout hospitals due to patients suspected of TB placed in general medical wards or patients unsuspected of TB being hospitalized for extended periods of time [23, 24]. These findings indicate a need to urgently investigate infection control measures and practices throughout hospitals including non-clinical areas and ensure all hospital staff members receive infection control training. Moreover, all staff should undergo routine TB symptom screening to identify and treat cases of TB in HCWs.

Pediatric wards are often considered to be low-risk areas as children with TB are less infectious than adults [25]. However, in this sample, we found HCWs who had a history of working in pediatric wards had an increased incidence of TB. Transmission of TB from family members or visitors to pediatric patients, HCWs, and visitors to pediatric wards has been reported [26–31]. In this setting with high TB incidence, it is possible that undiagnosed family members or visitors who spend extended periods of time in pediatric wards may expose HCWs to TB. HCWs working in pediatric wards may perceive themselves at low risk for TB and not adhere to infection control measures. While it was not directly assessed in this analysis, an infection control assessment was performed in each ward of each of the three study hospitals and none of the pediatric wards in these hospitals practiced key infection control measures such as maintaining appropriate ventilation through open windows or providing N95 respirators to staff. These findings suggest that infection control measures and practices in pediatric wards need to be urgently evaluated and addressed.

In this sample, the area with the greatest incidence of TB was working in the stores/workshop. While employees working in these areas move throughout the hospital, they spend less time in clinical areas than other HCWs. The risk in this group may indicate that infection control measures in these areas are inadequate due to a low perceived risk and poor to non-existent infection control measures due to the necessity to keep hospital supplies in a climate controlled environment. Transmission in this area may be occurring between staff working in these areas that may be exposed to TB in the community. Community exposure was not measured in this study. These findings indicate a need to further investigate infection control in non-clinical areas and to include non-clinical staff in infection control education. In addition, occupational health should include these non-clinical staff in TB symptom screenings to potentially identify cases amongst this group.

HIV is a well-known risk factor for TB and the risk for TB is estimated to be more than 20 times greater in those living with HIV [32]. In this sample HCWs living with HIV had the greatest incidence of TB disease. Reported HIV prevalence in South African HCWs is estimated to be between 11% and 16% [10, 11]. These data suggest that the HIV prevalence in HCWs is similar to that of the general population and that HIV-positive HCWs are at significant risk of TB and require protection against TB exposure in health care settings. While HCWs are offered and encouraged to know their HIV status; HIV counseling and testing and regular TB screening uptake remains low [22]. It is imperative for HCWs to know their HIV status and for those HCWs who are HIV-positive to be reassigned to low-risk areas when possible, provided antiretroviral therapy, and offered isoniazid preventive therapy (IPT) [33, 34].

Our comparison of TB incidence rates in this sample with the general population indicated that HCWs had a greater incidence of TB than the general population of the province and the districts where the hospitals are located. This further supports previous findings that HCWs, despite high levels of TB in the community, have greater risk of developing TB than the general population [35]. This suggests that current infection control measures and occupational health services are not completely effective at protecting HCWs in the workplace.

We acknowledge several potential limitations to this study. Data for this study were abstracted from occupational health clinic employee medical records and limited by the quality of the records. Due to missing data of interest, we excluded nearly 8% of medical charts. It is likely that some HCWs sought and received care outside of the hospital at private providers or hospitals and did not report their case of TB to the occupational health nurse and therefore, the incidence of TB in this sample may be underestimated. Similarly, a large proportion (74%) of HCWs did not have their HIV status documented in their medical charts suggesting that the HIV prevalence in this population may be underestimated. Many HCWs (15%), especially clinical staff, worked in more than one ward during the study period and this made it difficult to pinpoint where TB exposure may have occurred. However, our analysis included a multilevel multivariate model controlling for multiple wards to address this concern. It should be noted that the comparison of TB incidence rates among HCWs in this sample with the general population of KwaZulu-Natal is a crude comparison and does not account for potential confounding.

TB disease incidence in HCWs is currently used as an indicator of the effectiveness and quality of infection control measures [36]. While routine screening of HCWs for TB symptoms is relatively simple; the implementation of this can be challenging especially as screening of HCWs for TB is not mandatory and no official guidelines regarding screening HCWs for TB currently exist. For example, we found as part of a separate aim of this study that in 2010 only 19% of all HCWs across the hospitals were screened [22]. It is critical that at all levels of the health system, particularly at the hospital level, there be more coordinated efforts between occupational health and infection control programs to protect HCWs from TB, such as screen HCWs for TB, offer HIV counseling and testing, provide antiretroviral therapy and IPT for HCWs living with HIV, and provide effective and supportive care to HCWs with TB and HIV. HCWs should be educated and encouraged to be screened for TB routinely and efforts should be made to decrease the stigma associated with TB and HIV to make HCWs feel more comfortable about reporting these diseases to occupational health and seeking treatment.

## Conclusions

HCWs working in TB wards had a greater incidence of developing TB than HCWs without a history of working in TB wards. However, HCWs working in other hospital areas also had an increased incidence of TB, as were HCWs living with HIV. In addition, HCWs working in the three public hospitals in KwaZulu-Natal had a higher incidence of TB compared with that of the general population. While emphasis is placed on TB infection control measures in TB and MDR-TB wards, these findings strongly suggest that the risk of TB transmission to HCWs is occurring throughout hospital settings and infection control measures should be implemented and practiced throughout hospitals to protect HCWs from TB.
